# Aber-OWL: a framework for ontology-based data access in biology

**DOI:** 10.1186/s12859-015-0456-9

**Published:** 2015-01-28

**Authors:** Robert Hoehndorf, Karin Slater, Paul N Schofield, Georgios V Gkoutos

**Affiliations:** 1 Computational Bioscience Research Center, King Abdullah University of Science and Technology, 4700 KAUST, Thuwal, 23955-6900 Saudi Arabia; 2 Computer, Electrical and Mathematical Sciences & Engineering Division, King Abdullah University of Science and Technology, 4700 KAUST, Thuwal, 23955-6900 Saudi Arabia; 3 Department of Computer Science, Aberystwyth University, Llandinam Building, Aberystwyth, SY23 3DB UK; 4 Department of Physiology, Development & Neuroscience, University of Cambridge, Downing Street, Cambridge, CB2 3EG UK

**Keywords:** Ontology-based data access, Linked data, OWL

## Abstract

**Background:**

Many ontologies have been developed in biology and these ontologies increasingly contain large volumes of formalized knowledge commonly expressed in the Web Ontology Language (OWL). Computational access to the knowledge contained within these ontologies relies on the use of automated reasoning.

**Results:**

We have developed the Aber-OWL infrastructure that provides reasoning services for bio-ontologies. Aber-OWL consists of an ontology repository, a set of web services and web interfaces that enable ontology-based semantic access to biological data and literature. Aber-OWL is freely available at http://aber-owl.net.

**Conclusions:**

Aber-OWL provides a framework for automatically accessing information that is annotated with ontologies or contains terms used to label classes in ontologies. When using Aber-OWL, access to ontologies and data annotated with them is not merely based on class names or identifiers but rather on the knowledge the ontologies contain and the inferences that can be drawn from it.

## Background

A large number of ontologies have been developed for the annotation of biological and biomedical data, commonly expressed in the Web Ontology Language (OWL) [1] or an OWL-compatible language such as the OBO Flatfile Format [2]. Access to the full extent of knowledge contained in ontologies is facilitated by automated reasoners that can compute the ontologies’ underlying taxonomy and answer queries over the ontology content.

While ontology repositories, such as BioPortal [3] and the Ontology Lookup Service (OLS) [4], provide web services and interfaces to access ontologies, including their metadata such as author names and licensing, the list of classes and asserted structure, they do not enable computational access to the semantic content of the ontologies and the inferences that can be drawn from them. Access to the semantic content of ontologies usually requires further inferences to reveal the consequences of statements (axioms) asserted in an ontology; these consequences may be automatically derived using an automated reasoner. To the best of our knowledge, no reasoning infrastructure that supports semantically enabled access to biological and biomedical ontologies currently exists.

Here, we present Aber-OWL, a reasoning infrastructure over ontologies consisting of an ontology repository, web services that facilitate semantic queries over ontologies specified by a user or contained in Aber-OWL’s repository, and a user interface. Such an infrastructure can not only enable access to knowledge contained in ontologies, but crucially can also be used for semantic queries over data annotated with ontologies, including the large volumes of data that are increasingly becoming available through public SPARQL endpoints [5]. Allowing access to data through an ontology is known as the “ontology-based data access” paradigm [6,7], and can exploit formal information contained in ontologies to:

- identify possible inconsistencies and incoherent descriptions [8],
- enrich possibly incomplete data with background knowledge so as to obtain more complete answers to a query (e.g., if a data item referring to an organism has been characterized with multiple findings that together constitute a disease, then the data item can be returned when querying for the disease even in the absence of it being explicitly declared in a database) [6,9],
- enrich the data schema used to query data sources with additional information (e.g., by using a class in a query that is an inferred super-class of one or more classes that are used to annotate data items, but the class itself is never used to characterize data) [6], and
- provide a uniform view over multiple data sources with possibly heterogeneous, multi-modal data [6,7].

To demonstrate how Aber-OWL can be used for ontology-based access to data, we provide a service that performs a semantic search over PubMed and PubMed Central articles using the results of an Aber-OWL query, and a service that performs SPARQL query extension so that the results of Aber-OWL queries can be used to retrieve data accessible through public SPARQL endpoints. In Aber-OWL, following the ontology-based data access paradigm [6,7], we specify the features of the relevant information on the ontology- and knowledge level [10], and retrieve named classes in ontologies satisfying these condition using an automated reasoner, i.e., a software program that can identify whether a class in an ontology satisfies certain conditions based on the axioms specified in an ontology.

Subsequently, we embed the resulting information in database, Linked Data or literature queries.

Aber-OWL can be accessed at http://aber-owl.net. The Aber-OWL software is freely available at https://github.com/reality/SparqOWL can be installed locally by users who want to provide semantic access to their own ontologies and support the use of their ontologies in semantic queries.

## Methods

### Aber-OWL

The Aber-OWL software can be configured with a list of URIs that contain ontology documents (i.e., OWL files) and employs the OWL API [11] to retrieve the ontologies that are to be included in the repository. For each ontology document included in the repository, the labels and definitions of all classes contained within the ontology (as well as of all the ontologies it imports) are identified based on OBO Foundry standards and recommendations: we use the rdfs:label annotation property to identify class labels for each ontology (as well as of all the ontologies it imports), and we employ the *definition* (http://purl.obolibrary.org/obo/IAO_0000115) annotation property, defined in the Information Artifact Ontology, to identify the text definitions of a class.

Labels of the classes occurring in each ontology, as well as of all the ontologies it imports, are stored in a trie (prefix tree). The use of a trie ensures that class labels can be searched efficiently, for example when providing term completion recommendations.

Upon initiating the Aber-OWL web services, we classify each ontology using the ELK reasoner [12], i.e., we identify the most specific sub- and super-classes for each class contained in the ontology using the axioms contained within it. The ELK reasoner supports the OWL EL profile [13] and ignores ontology axioms that do not fall within the OWL EL subset. The benefit of using the OWL EL profile is the support for fast, polynomial-time reasoning, and the OWL EL subset is a suitable dialect for a large number of biomedical ontologies [14]. While we currently use ELK for the Aber-OWL infrastructure, it is possible for a user to install an Aber-OWL server that employs different OWL reasoners, such as HermiT [15] or Pellet [16], using the standard reasoner interface of the OWL API.

Querying is performed by transforming a Manchester OWL Syntax [17] query string into an OWL class expression using the OWL API and then Aber-OWL’s short-form provider is employed to provide the mappings of the OWL class and the property URIs to the class and property labels. If this transformation fails (i.e., when the query string provided is not a valid OWL class expression within the ontology being queried), an empty set of results is returned. If the transformation succeeds, the ELK reasoner is used to retrieve sub-, super- or equivalent classes of the resulting OWL class expression. The type of query (sub-class, super-class, or equivalent class) is specified by the user and defaults to a sub-class query. Queries in which the URL of the ontology document is not specified are delegated to all ontologies in Aber-OWL’s repository. Consequently, results may be returned from multiple ontologies. If an ontology URL is specified as part of a query using the Aber-OWL webservices but the ontology it corresponds to is not available within Aber-OWL’s repository, an attempt is made to retrieve the ontology from the URL, which is then classified and then the query results over the classified ontology are returned to the user. Should this process fail, an empty set of results is returned.

The results of an Aber-OWL query are provided in JSON format [18] and consist of an array of objects containing information about the ontology classes satisfying the query: the URI of the ontology document queried, the IRI of the ontology class, the class label and the definition of the class. Detailed documentation of the web services is available at the Aber-OWL web site.

We implemented a web server that can be used to access Aber-OWL’s ontology repository and reasoning services. The web server features a JQuery-based [19] interface and uses AJAX [20] to retrieve data from the Aber-OWL web services.

### Aber-OWL: PubMed

Aber-OWL: PubMed is built on top of the Aber-OWL reasoning infrastructure. It employes the Aber-OWL reasoning infrastructure to resolve a semantic query formulated in Manchester OWL Syntax and retrieve a set of named classes that satisfy the query. We use the results to perform a Boolean textual search over a corpus of articles.

We use the Apache Lucene framework [21] to create a fulltext index of all titles and abstracts in MEDLINE/PubMed 2014 [22], and all fulltext articles in PubMed Central [23]. Before indexing, every text is processed using Lucene’s English language standard analyzer which tokenizes and normalises it to lower case as well as applies a list of stop words.

For a user-specified query in Manchester OWL syntax, we construct a Lucene query string from the set of class descriptions returned from the Aber-OWL services. In particular, we concatenate each class label using Lucene’s OR operator. As a result, the Lucene query will match any article (title, abstract or fulltext) that contains a label of a class satisfying the semantic query. Although we use Lucene’s relevance scoring of matches in documents and return documents in order of decreasing relevance, queries for high-level classes will often result in very unspecific search results due to the large number of possible labels that are considered in the query. One possibility to make queries more specific is to conjunctively perform multiple semantic queries by providing more than one query in Manchester OWL syntax.

### Aber-OWL: SPARQL

Data in biology is commonly annotated to named classes in ontologies, identified through a URI or another form of identifier that usually directly maps to a URI. Pieces of data may refer to genes and proteins, text passages, measurements and other observations, and can be presented in multi-modal form as text, formal statements, images, audio or video recordings. This information is increasingly being made available as Linked Data through publicly available SPARQL endpoints [5,24].

To semantically access ontology-annotated data contained in datasets available through public SPARQL endpoints, we provide a service which extends the SPARQL language with syntax which allows the user to include Aber-OWL resultsets within the query. This comprises of a list of class URIs returned by Aber-OWL, which can then be used to match data in the SPARQL endpoint. SPARQL query expansion is implemented using the PHP SPARQL library [25] and is available both as a web service and through a web interface that can be accessed through Aber-OWL’s main web site.

## Results

### Aber-OWL

The Aber-OWL framework can be used to retrieve all super-classes, equivalent classes or sub-classes resulting from a Manchester OWL Syntax query. The classes are retrieved either from a specific ontology in Aber-OWL’s ontology repository, from all ontologies in the repository, or from a user-specified ontology that can be downloaded from a specified URI. In our installation of Aber-OWL at http://aber-owl.net, the complete library of OBO ontologies [26] is imported as well as several user-requested ontologies.

Using our web server, any ontology in Aber-OWL’s ontology repository can be queried and the results subsequently displayed. Furthermore, following execution of any Aber-OWL query, the web interface provides the means to use the query in Aber-OWL: PubMed so as to search and retrieve relevant biomedical literature, or in Aber-OWL: SPARQL to construct a query for data annotated to one of the resulting classes.

### Ontology-based access to literature

Aber-OWL: PubMed enables ontology-based semantic access to biomedical literature. It combines the information in biomedical ontologies with automated reasoning to perform a literature query for all things that can be inferred from a class description within one or more ontologies. For example, the complex cardiac malformation, *Tetralogy of Fallot*, is made up of a set of four characteristic defects in different components of the heart, one of which is a ventricular septal defect. A query for the class ’ventricular septal defect’ will return articles in which, among others, ’tetralogy of fallot’ is mentioned due to ’tetralogy of fallot’ being inferred to be a subclass of ’ventricular septal defect’ in the Uberpheno [27] and Human Phenotype [28] ontologies. Since Aber-OWL uses an automated reasoner to identify subclasses, this information does not have to be asserted in the ontology but rather is implied by the ontology’s axioms.

Aber-OWL: PubMed can also perform more complex queries, such as for articles containing mentions of subclasses of part_of some ’apoptotic process’ and part_of some regulation, and articles mentioning regulatory processes that are a part of apoptosis will be returned. Such queries are only possible through the application of automated reasoning over the knowledge contained in the biomedical ontologies, and go beyond the state of the art in that they enable a genuinely *semantic* way of accessing biomedical literature based on the knowledge contained in the ontologies.

Finally, Aber-OWL: PubMed can also be used to identify co-occurrences of multiple Aber-OWL queries. For example, a conjunctive combination of two sub-class queries, one for ’ventricular septal defect’ and another for part_of some heart, will return articles that contain references to both parts of the heart (such as the aorta) and particular types of ventricular septal defects, e.g., muscular or membranous defects, as well as complex phenotypes such as the Tetralogy of Fallot.

Aber-OWL: PubMed is accessible through a basic web interface at http://aber-owl.net/aber-owl/pubmed/ in which queries can be executed, the articles satisfying the queries will be displayed, and matching text passages in the title, abstract or fulltext will be highlighted. Furthermore, Aber-OWL: PubMed can be accessed through web services and thereby can be embedded in web-based applications.

### Ontology-based access to linked data

Aber-OWL: SPARQL provides semantic access to Linked Data by expanding SPARQL queries with the results returned by an Aber-OWL query. Query expansion is performed based on SPARQL syntax extended by the following construct:


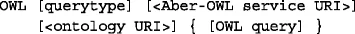


For example, the query


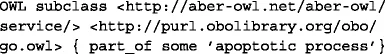


will return a set of class URIs that satisfy the query part_of some ’apoptotic process’ in the Gene Ontology (GO) [29], and the results will be embedded in the SPARQL query. For this purpose, the OWL statement is replaced by the Aber-OWL: SPARQL service with a set of class URIs. There are two main forms in which the OWL statement can be embedded within a SPARQL query. The first is the VALUES form in which the results of the OWL query are bound to a variable using the SPARQL 1.1 VALUES statement. For example,


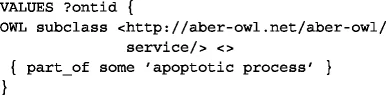


will bind the ontology URIs resulting from the OWL query (part_of some ’apoptotic process’) to the SPARQL variable ?ontid. The second form in which the OWL statement is useful is in the form of a FILTER statement. For example, the query


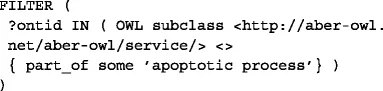


will filter the results of a SPARQL query such that the values of ?ontid must be in the result list of the OWL query.

As many SPARQL endpoints use different URIs to refer to classes in ontologies, we have added the possibility to re-define prefixes for the resulting ontology classes such that they match the IRI scheme used by a particular SPARQL endpoint. When this feature is used, the class IRIs resulting from an OWL query will be transformed into a prefix form similar to the format used in the OBO Flatfile Format [2], and the appropriate prefix definition will be added to the SPARQL query if it has not been defined in the query already. For example, the UniProt SPARQL endpoint (http://sparql.uniprot.org) uses the URI pattern http://purl.uniprot.org/go/<id> to refer to Gene Ontology classes, the EBI BioModels endpoint uses http://identifiers.org/go/<id>, while the URI policy of the OBO Foundry [30] specifies that the URI pattern http://purl.obolibrary.org/obo/GO_<id> should be used. The latter URI scheme is the one employed by Aber-OWL since this is the authoritative URI provided in the ontology document. Using the prefix format will transform the results of the Aber-OWL query from URIs into strings of the type GO:<id> and the appropriate prefix to the SPARQL query (i.e., PREFIX GO: < http://purl.obolibrary.org/obo/GO_ > will be added. Changing this prefix definition statement to PREFIX GO: < http://purl.uniprot.org/go/ > will effectively rewrite the URIs so that they can be used in conjunction with the URI scheme employed by the UniProt SPARQL endpoint. Alternatively, the SPARQL query can employ a dedicated mapping service, possibly in the form of a SPARQL endpoint with access to sameAs statements, to convert between URI schemes used in different places.

#### Use case: find all human proteins associated with a ’part of apoptosis’ in UniProt

**Figure 1:**
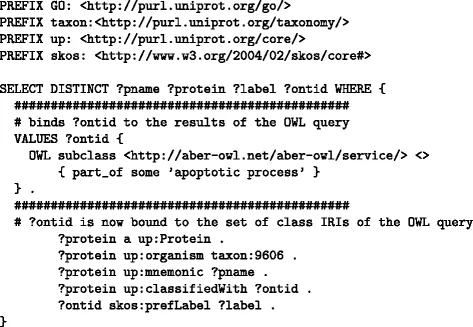
**A query for all human proteins annotated to a part of apoptosis.** The query is executed against the UniProt SPARQL endpoint at http://sparql.uniprot.org. To rewrite the URI scheme used by UniProt for GO classes to the URI scheme returned by Aber-OWL, Aber-OWL: SPARQL must be used with the prefix rewriting option set to true.

We can demonstrate the possibilities of using the Aber-OWL: SPARQL query expansion service by retrieving all human proteins in UniProt [31] annotated to part_of some ’apoptotic process’. To achieve this goal, we use the SPARQL 1.1 VALUES statement to bind the results to a variable ?ontid, and then we can use this variable in the SPARQL query to retrieve all human proteins with a Gene Ontology annotation in ?ontid. The query is shown in Figure 1.

As UniProt uses different URIs for GO classes than those returned by Aber-OWL (which are based on the officially endorsed URIs by the OBO Foundry and the Gene Ontology Consortium), the URIs have to be rewritten for the query to succeed. In particular, in Aber-OWL: SPARQL, an option must be activated to rewrite URIs into a “prefix form” (i.e., URIs of the type http://purl.obolibrary.org/obo/GO_0008150 would be rewritten to GO:0008150), and the SPARQL PREFIX declaration will redefine the prefix to match the URI scheme used in the UniProt SPARQL endpoint.

#### Use case: search GWAS Central for genes and markers significantly involved in ventricular septal defects

**Figure 2:**
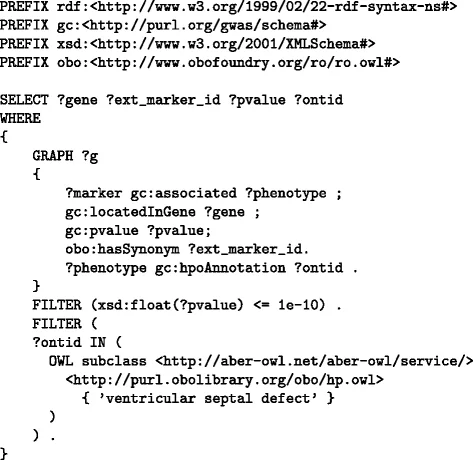
**A SPARQL query for markers significantly associated with** ***ventricular septal defect*** **.** The query is executed against the GWAS Central SPARQL endpoint at http://fuseki.gwascentral.org/query.html.

We can also utilize the Aber-OWL infrastructure for more powerful queries that use inference over the ontology structure and utilize the results in a SPARQL query. For example, we can use Aber-OWL: SPARQL to query GWAS Central [32] for markers that have been identified in GWAS studies as significant for ventricular septal defects (Figure 2).

Using the Human Phenotype Ontology (HPO) [28] and the definitions that have been developed for the HPO [33], we can identify that a Tetralogy of Fallot involves a particular type of ventricular septal defect. In particular, according to the axioms contained in the HPO, a Tetralogy of Fallot condition can be inferred from the phenotypes *ventricular septal defect*, *overriding aorta*, *pulmonary valve stenosis* and *right ventricular hypertrophy*. Importantly, no explicit subclass relation between these four key phenotypes and *Tetralogy of Fallot* is asserted in the HPO. Therefore, reasoning is required to retrieve *Tetralogy of Fallot* as a subclass of either of these four or a combination of these four, phenotypes. Similarly, OWL reasoning over the ontology axioms is required to retrieve data annotated to Tetralogy of Fallot when querying for any of the four phenotypes. The queries can also be made more precise by explicitly asking for a condition in which all four of the Tetralogy of Fallot phenotypes must be satisfied: subclasses of ’overriding aorta’ and ’ventricular septal defect’ and ’pulmonic stenosis’ and ’right ventricular hypertrophy’ will specifically retrieve the Tetralogy of Fallot condition, including specific sub-types of Tetralogy of Fallot in the HPO.

## Discussion

### Comparison to related work

BioPortal [3], the Ontology Lookup Service (OLS) [4] and Ontobee [34] are amongst the most widely used ontology repositories in biology. These portals offer a user interface for browsing ontologies and searching for classes based on the class label (or synonym). They also provide web services that enable programmatic access to the ontologies contained within them, and at least when ontologies are made available in a pre-reasoned form (i.e., when axioms that have been inferred by an automated reasoner were incorporated in the ontology file), they also provide access to some of the knowledge that can be derived from the ontologies’ axioms. However, neither BioPortal, Ontobee nor OLS allow access to additional knowledge that can be derived from the ontologies in the repositories through deductive inference (i.e., queries). Aber-OWL, on the other hand, provides semantic access to biological ontologies through an automated reasoner, and can infer a ontology’s class hierarchy as well as answer queries using deductive inference. However, while Aber-OWL provides a reasoning infrastructure and reasoning services for ontologies, it does not aim at replacing ontology repositories and the user experience they provide. In the future, we intend to integrate Aber-OWL more closely with other ontology repositories so that the additional information and user-interface widgets provided by these repositories can be combined with the reasoning infrastructure provided by Aber-OWL.

A related software is OntoQuery [35], which is a web-based query interface for ontologies that uses an OWL reasoner. It can be used to provide an interface for a single ontology using an OWL reasoner, but does not support use of multiple ontologies or access through web interfaces.

The Logical Gene Ontology Annotations (GOAL) [36] outlines an approach to access data annotated with ontologies through OWL reasoning. For this purpose, GOAL constructs a custom knowledge base integrating both the ontology and the annotations, and then uses an OWL reasoner to answer queries over this combined knowledge base. However, GOAL uses exactly one ontology, specifically built to incorporate the data queried (mouse phenotypes) as a part of the OWL ontology so that a reasoner can be used to query both, the ontology and its annotations. Aber-OWL, on the other hand, is a general framework and does not require changes to existing ontologies. Instead, Aber-OWL distinguishes between reasoning on the ontology level and retrieval of data annotated with ontologies.

There are multiple projects that generate and maintain large linked datasets in the life sciences, and almost all these datasets and repositories utilize ontologies to characterize the data. For example, the Linked Life Data project [37] integrates 25 biological databases and provides access to them through a single SPARQL endpoint; the Bio2RDF project [24] is a community effort to make biological and biomedical databases accessible using Semantic Web technologies, and Bio2RDF release 3 already integrates 35 biological datasets; the Open PHACTS project [38] is the result of a collaboration between academic institutes, publishers, and pharmaceutical companies, and developed a large integrated dataset accessible through SPARQL endpoints; and the European Bioinformatics Institute (EBI) recently released several of their databases as RDF datasets accessible through public SPARQL endpoints [5]. Each of these projects provide ontology-annotated datasets and allow some inferences over ontologies and their annotations. In particular, most RDF endpoints in these projects support RDFS entailment [39], and the Linked Life Data endpoint, based on BigOWLIM [40], supports rules which implement a subset of OWL entailment. With regard to the possible inferences over ontologies, Aber-OWL differs from these projects in that it separates reasoning over ontologies from retrieval of ontology-annotated data, allows rich inferences over OWL ontologies based on the OWL-EL profile [13], and allows access to reasoning over ontologies through the Manchester OWL syntax [17].

Additionally, the use of Aber-OWL: SPARQL differs in three key points from the use of basic access to ontology-annotated data through SPARQL alone:

1. Aber-OWL: SPARQL provides access to the semantic content of ontologies even when the ontologies are not available through the SPARQL endpoint that contains the ontology-annotated data.
2. Aber-OWL: SPARQL provides access to the inferred ontology structure instead of the asserted structure, even when no OWL entailment regime is activated in a SPARQL endpoint, or when the OWL entailment regime does not support the OWL-EL profile.
3. Aber-OWL: SPARQL enables complex queries formulated in Manchester OWL syntax.

In particular, (1) the ontologies used for annotation are not commonly accessible through the same SPARQL endpoint as the actual annotated data. If the SPARQL endpoint supports query federation (using the SPARQL SERVICE block), this problem can usually be resolved if the ontology is available at some place (such as BioPortal) through another SPARQL endpoint. However, in some application settings, a query expansion service may be more efficient than query federation. More importantly, however, (2) Aber-OWL: SPARQL provides access to the structure of an ontology as it is inferred by an OWL reasoner. To achieve a similar outcome using plain SPARQL, the SPARQL endpoint containing the ontology must have an OWL entailment regime [39] activated; otherwise, only the asserted structure of an ontology is available for queries. We know of no SPARQL endpoint in the biomedical domain currently holding ontologies and simultaneously using an OWL entailment regime; in particular, neither BioPortal nor Ontobee or the OLS currently make use of any kind of OWL entailment. While the first two points can in principle be addressed by applying Semantic Web technologies, queries would still have to be formulated in SPARQL syntax. (3) Aber-OWL: SPARQL uses the Manchester OWL syntax to formulate queries, and Manchester OWL syntax is widely used by ontology developers and users as it is closer to a human-readable sentence and therefore easier to access than other ways of expressing OWL.

Several tools and web servers utilize ontologies or structured vocabularies for the retrieval of articles from PubMed or PubMed Central. For example, GoPubMed [41] classifies PubMed articles using the GO [29] and the Medical Subjects Heading thesaurus [42]. However, GoPubMed uses only a limited number of ontologies, and while GoPubMed uses the asserted structure of the ontologies, it does not use the knowledge contained within the ontologies’ axioms. Aber-OWL: PubMed, on the other hand, can utilize the knowledge contained in any ontology to perform basic searches in PubMed abstracts and fulltext articles in PubMed Central.

### Limitations

A main limitation of Aber-OWL: PubMed lies with the absence of a specialized entity recognition method to identify occurrences of ontology class labels in text. In particular, for ontologies such as the GO that use long and complex class names, specialized named entity recognition approaches are required to identify mentions of the GO terms in text [43,44]. Furthermore, Aber-OWL: PubMed currently uses only the rdfs:label property of classes and properties in ontologies to retrieve literature documents, but ignores possible synonyms, alternative spellings or acronyms that may be asserted for a class. In the future, we will investigate the possibility of adding more specialized named entity recognition algorithms to Aber-OWL: PubMed for specific ontologies.

Another limitation lies in Aber-OWL’s interface. Aber-OWL: PubMed’s web-based interface is not a complete text retrieval system but rather demonstrates the possibility of using ontology-based queries for retrieving text and can be used to aid in query construction. We envision the main use of Aber-OWL: PubMed in the form of its web services that can be incorporated in more complete and more complex text retrieval systems such as GoPubMed or even PubMed itself.

### The need for improved interoperability between biomedical ontologies

The full benefit of a reasoning infrastructure over multiple ontologies can be realized when these ontologies are “interoperable”. While interoperability between biomedical ontologies has been extensively discussed [8,26,45,46], we can nevertheless identify several shortcomings through the use of Aber-OWL. Firstly, ontology class names and relation names are not standardized. For example, the current library of ontologies included in Aber-OWL uses several different names (and URIs) for the part-of relation, including part_of, part-of, ’part of’ and PartOf. While each relation is usually consistently applied within a single ontology, the use of different URIs and labels for the same relation leads to difficulties when utilizing more than one ontology. The non-standardized use of relation names is particularly surprising as the OBO Relation Ontology [45] aimed to achieve the goal of using standard relations and common relation names almost 10 years ago. One possible explanation for the observed heterogeneity is that the lack of tools and an infrastructure that could efficiently utilize the information in one or more ontology has made it less of a priority for ontology developers to focus on these aspects of interoperability.

Furthermore, using the Aber-OWL infrastructure, potential problems in ontologies can be identified. For example, we could identify, and subsequently correct, three unsatisfiable classes in the Neuro Behavior Ontology [47] resulting from changes in the ontologies it imports. These problems are not easily detectable; moreover, they require the use of reasoning over more than one ontology, as well as frequent re-classifications. These tasks are vital for the effects that a change in one ontology has on other ontologies to be detected.

## Conclusions

With the Aber-OWL services, we propose to separate the processing of knowledge in ontologies and the retrieval of data annotated with these ontologies. Aber-OWL provides a reasoning infrastructure that can be queried either through its web interface or its web services, and a set of classes that satisfy a specified condition is returned. These sets of classes can then be used to retrieve data annotated with them, text that contains their label, or from a corpus of text or a formal data resource that references them. As such, Aber-OWL provides a framework for automatically accessing information that is annotated with ontologies or contains terms used to label classes in ontologies. When using Aber-OWL, access to the information in ontologies is not merely based on class names or identifiers but rather on the knowledge the ontologies contain and the inferences that can be drawn from it. This also enables the use of knowledge- and ontology-based access to data [6,7]: data of interest is specified on the knowledge- or ontology-level [10], and all possible classes that satisfy such a specification are inferred using an automated reasoner. The results of this inference process are then used to actually retrieve the data without the need to apply further inference.
